# Satisfaction with a digital support tool targeting alcohol consumption: perspectives from participants in a randomized control trial

**DOI:** 10.1093/alcalc/agad070

**Published:** 2023-10-30

**Authors:** Elizabeth S Collier, Jenny Blomqvist, Marcus Bendtsen

**Affiliations:** Department of Health, Medicine and Caring Sciences, Linköping University, Linköping, Sweden; Division of Bioeconomy and Health, Department of Material and Surface Design, RISE Research Institutes of Sweden, Stockholm, Sweden; Department of Health, Medicine and Caring Sciences, Linköping University, Linköping, Sweden; Department of Health, Medicine and Caring Sciences, Linköping University, Linköping, Sweden

**Keywords:** alcohol, intervention design, experiences, behavioural change, feedback

## Abstract

**Aim:**

Intervention design may be improved through evaluating the feedback from those who have been exposed to such interventions. As such, here the perspectives of the intervention group from a recent randomized control trial investigating the effectiveness of a digital alcohol intervention, in terms of perceived suitability and usefulness of the support tool they engaged with, were investigated.

**Methods:**

Respondents (N=475; 45% of the intervention group) answered five quantitative questions addressing user experience, completed the 10-item System Useability Scale, and were offered the opportunity to write free-text feedback. Quantitative measures were analysed using ordinal and linear regression with baseline characteristics as predictors, and free-text responses were evaluated using content analysis.

**Results:**

Overall, respondents were positive towards the intervention in terms of it fitting their needs, the usefulness of the tools included, and the usefulness of text message content. The intervention was perceived as more helpful by respondents with lower total weekly alcohol consumption, higher self-reported confidence in their ability to reduce their drinking, and the perceived importance there of, at baseline. The free-text comments revealed the value of reminders as prompts to reflect on one’s own drinking behaviour. Nonetheless, criticisms of the intervention were voiced, primarily highlighting the repetitive nature of the reminders and the lack of individuation in advice. Some also feltlike the intervention was impersonal and targeted only a specific drinking pattern.

**Conclusions:**

Experiences of the intervention group in this trial were generally positive, though there may be demand for more individualised, targeted intervention design.

## Teaser

**Short Summary:** Perspectives from participants in a recent parallel group randomized effectiveness trial of a digital alcohol intervention are investigated. Experiences were generally positive, and the efficacy of the behavioural change mechanisms underpinning the intervention design are supported. Nonetheless, more individualized, targeted intervention design may be beneficial in the future.

## Introduction

Regular consumption of alcohol is associated with an increased risk of several non-communicable diseases (Wood *et al*. 2018), as well as harm to others (Andréasson *et al*. 2015; Stanesby *et al*. 2018) and wider society (Jarl *et al*. 2008; Nayak *et al*. 2019). Nonetheless, drinking remains prevalent in many societies, including Sweden where, according to national guidelines, ~30% of the adult population report unhealthy alcohol use (Guttormsson 2022). In Sweden, this is currently defined as drinking ˃9 (female) or 14 (male) standard drinks (12 g of alcohol) of alcohol per week (total weekly consumption) or drinking ˃4 (female) or 5 (male) standard drinks on a single occasion at least once a month (heavy episodic drinking). Since Sweden already has relatively strict policies in place regarding alcohol (a state sales monopoly—meaning that alcohol for at-home consumption can only be purchased at the designated, state-owned and -operated store—and high alcohol taxes), there is a role to be played by other actors in offering support to those seeking to reduce their alcohol consumption. For example, offering brief alcohol interventions in primary care settings can be an effective approach (O'Donnell *et al*. 2014). However, at the time of writing, the number of individuals visiting primary healthcare receiving advice and feedback relating to lifestyle behaviours in Sweden remains low (<6%) and advice related to drinking is given less often relative to other factors such as diet and exercise (Swedish National Board of Health and Welfare 2022).

Since internet access and mobile phone ownership are near-ubiquitous in many high-income countries, including Sweden, interventions that can be delivered digitally via mobile phone are of increasing interest and relevance. Delivery of intervention content can vary from reminders or prompts sent via email or text message to development of downloadable applications (apps) including tracking and planning components, or combinations of these approaches. A Cochrane review of trials testing digital alcohol interventions indicated favourable outcomes in terms of both total weekly alcohol consumption and the frequency of heavy episodic drinking (Kaner *et al*. 2017). Such digital interventions could also offer additional benefits, including broader accessibility, reduced stigma due to increased perceived anonymity, and the potential to conveniently provide on-going, repeated support (Kaner *et al*. 2017; Boumparis *et al*. 2019). However, the evidence in favour of employing digital alcohol interventions is not yet conclusive or without fault (Palmer *et al*. 2018; Bendtsen *et al*. 2022) and establishing the most effective approaches to designing and offering these is not trivial (Crane *et al*. 2018; Bendtsen and McCambridge 2019). This may be unsurprising, given the difficulty and complexity of changing health behaviours such as alcohol consumption, and the heterogeneity amongst individuals seeking help for their drinking behaviour.

Developing digital intervention tools that are of maximal value to those who need them can be aided by evaluating the experiences of those who have engaged with them. Understanding the experiences of trial participants can also provide insight into the mechanisms that may be most relevant for supporting change, or evaluating whether changes in behaviour are associated with intervention design in the intended manner. An earlier investigation of user-experience amongst the intervention group in a randomized control trial targeting alcohol consumption indicated that participation in the study prompted reflection over their drinking, in turn reducing consumption (Müssener *et al*. 2018). Therefore the present study assesses feedback from the intervention group from a recent two-arm, single-blind, parallel group randomized effectiveness trial of a digital alcohol intervention, the results of which have been reported elsewhere (Bendtsen *et al*. 2022). The trial was prospectively registered (ISRCTN48317451) and a trial protocol including a statistical analysis plan was made available prior to trial commencement (Bendtsen and McCambridge 2019). The objectives were to:

- Assess the perceived usefulness and suitability of the intervention according to those randomized to the intervention group during the main trial.
- Estimate associations between individuals’ baseline characteristics and satisfaction with the intervention.
- Evaluate individual’s free-text descriptions of what they perceived as positive and negative about the intervention.

## Materials and methods

Ethical approval for the study was received on 2018-06-11 by the Regional Ethical Committee in Linköping, Sweden (Dnr 2018/417–31). The target population was Swedish adults seeking help online to reduce their alcohol consumption. Individuals were required to be at least 18 years of age, have access to a mobile phone, and have unhealthy alcohol use according to Swedish guidelines: drinking ˃9 (female) or 14 (male) standard drinks (12 g of alcohol) of alcohol per week (total weekly consumption) or drinking ˃4 (female) or 5 (male) standard drinks on a single occasion at least once a month (heavy episodic drinking). All study materials were in Swedish.

### Intervention

**Figure 1:**
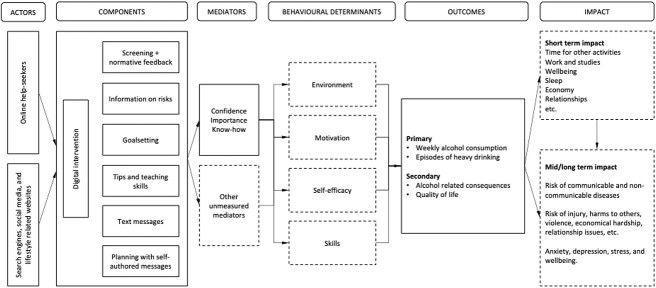
Logic model of the digital alcohol intervention, describing actors, intervention components, determinants of behaviour, short- and long-term effects, and impacts

In Fig. 1, a logic model of the digital alcohol intervention is presented describing actors, intervention components, determinants of behaviour, short- and long-term effects, and impacts. Designed based on social cognitive theories of health behaviour (Conner and Norman 2005), the digital alcohol intervention targets improving motivation and self-efficacy, as well as teaching new skills and addressing environmental constraints—which are understood to improve the likelihood of successful behaviour change (Fishbein *et al*. 2001), including for changing one's drinking.

To manipulate these important health determining factors, the intervention was delivered to participants via their mobile phones as a toolbox with six modules. The content of these modules was anchored in state-of-the-art empirical evidence for which active ingredients are effective for changing behaviour leading to reduced alcohol consumption, including behaviour substitution, problem solving, goalsetting, review of behavioural goals, self-monitoring, normative feedback, and understanding consequences of alcohol consumption (Michie *et al*. 2012; Garnett *et al*. 2018).

The core element of the intervention was a text message sent to participants each Sunday afternoon. The message included a prompt to self-monitor one's current alcohol consumption and a link to a web-based screening tool. The screening tool assessed past week's consumption, and participants were subsequently given access to the toolbox with six modules. In brief, these modules consisted of:

(i) Normative feedback on past week's consumption based on age and sex and classification as risky drinker.
(ii) Information about some of the risks from drinking alcohol, including risk of disease, how it may affect children in proximity, injuries, and traffic accidents.
(iii) A goalsetting tool with feedback on previously set goals and a timeline showing consumption over time.
(iv) Tips and teaching of skills which participants could use in their everyday life to immediately reduce their drinking, including tasks designed to make participants reflect on their behaviour.
(v) Text messages with tips, skills, and reflection tasks could be turned on at participants' discretion which were then sent to participants' mobile phone throughout the week.
(vi) A planning tool which allowed participants to write messages to themselves, which were sent to them at regular intervals throughout the week.

### Measures

At the 4-month follow-up, participants in the intervention group were asked the following five questions, created specifically to assess experience of the intervention:

(i) Overall, how well-suited do you believe that the support was to your needs?
(ii) (1 = ‘Not very well’ to 5 = ‘Very well’).
  (a) Please leave a comment describing your needs and how the intervention matched or did not match them (free text).
(iii) Do you believe that the content of the support tool would be helpful for people that want to reduce their consumption?
(iv) (1 = ‘Not very helpful’ to 5 = ‘Very helpful’)
(v) Do you believe that the content in the text messages would be helpful for people that want to reduce their consumption?
  (1 = ‘Not very helpful’ to 5 = ‘Very helpful’)
(vi) If you were to continue using the support, for how much longer would you want to use it?
  (a) I would use it for one to two more months
  (b) I would use it for three to six more months
  (c) I would use it for ˃6 months
  (d) I do not want to use it any more
  (e) I do not know
(vii) Would you recommend this intervention to a friend who expresses a wish to reduce their alcohol consumption?
  (a) Yes
  (b) No
  (c) I do not know

Additionally, the System Useability Scale (SUS) was administered, which consists of the following 10 agree/disagree statements (1 = strongly agree; 5 = strongly disagree).

- I think that I would like to use this system frequently.
- I found the system unnecessarily complex.
- I thought the system was easy to use.
- I think that I would need the support of a technical person to be able to use this system.
- I found the various functions in this system were well integrated.
- I though there were too much inconsistency in this system.
- I would imagine that most people would learn to use this system very quickly.
- I found the system very cumbersome to use.
- I felt very confident using the system.
- I needed to learn a lot of things before I could get going with the system.

A total SUS score was calculated by first converting responses by either subtracting 1 from the response (for odd numbered items) or subtracting the response from 5 (for even numbered items), summing up the converted responses, and finally multiplying by 2.5 to create a score from 0 to 100.

### Data analysis

Descriptive statistics (median and interquartile range, IQR) were calculated for the responses to questions 1 through 5 and SUS scores. Questions 1–3 were analyzed using ordinal regression with baseline characteristics as covariates to assess whether experience of the tool and its component parts were associated with respondent baseline characteristics. The ordinal models were parameterized to estimate odds ratios (OR) of responding more in agreement to the three questions. We also used linear regression to study differences in SUS scores with respect to respondents’ baseline characteristics. For all models Bayesian inference was used to estimate posterior distributions of coefficients, using Student-t priors centred at 0 with 3 degrees of freedom and a scale of 2.5 for all parameters (half-Student-t priors for error terms). Posterior medians are reported as point-estimates of associations along with 95% compatibility intervals (CI) defined by the 2.5% and 97.5% percentiles of the posterior distributions.

The free-text responses to question 1 were initially read in full by one author (ESC) to assess the general scope of the dataset. The comments were then re-read and assessed according to whether a positive view negative view was expressed, and recurring keywords and phrases were identified (content analysis). The vast majority of comments either described aspects of the intervention in either a positive or negative light (or sometimes both if multiple aspects of the intervention were discussed by a respondent) but almost never neutrally. Comments leaning neither positive nor negatively typically did not describe the individuals’ perspective on the intervention itself. The comments were coded according to the initial list of keywords and phrases, leading to a refined list of categories within each of positive and negative-leaning comments. A second author (JB) independently read the responses, listing key words, phrases, and concepts. The two lists were compared for discrepancies, and category descriptions were refined and adjusted. Following agreement on the categories and sub-categories perceived as positive and negative, ESC and JB coded the full dataset together to ensure consensus. Translated, anonymized quotes are provided to exemplify the (sub)categories.

## Results

### Response rate

The demographics of the full intervention group and those who responded to the follow-up questionnaire are shown in Table 1. For question 1, there were 475 respondents, whilst for questions 2–5 there were 479 respondents.

**Table 1:** Baseline characteristics of total participants in the intervention arm of the trial, and the subset of responders to the experiences survey.

	**Intervention group** *n* = 1063	**Responders** *n* = 475^a^
Total weekly consumption past week, median units consumed (IQR)	17 (10;25)	16 (10;24)
Episodes of heavy drinking past month, median episode frequency where 4+ drinks were consumed (IQR)	6 (4;10)	6 (3;10)
		
Age, median (IQR)	45 (35;55)	48 (40;57)
Sex, *n* (%)		
Female	612 (58%)	275 (58%)
Male	451 (42%)	200 (42%)
Household characteristics, *n* (%)		
Not living alone with kids (Living with somebody with kids)	383 (36%)	170 (36%)
Not living alone no kids (Living with somebody without kids)	267 (25%)	130 (27%)
Living alone with no kids (Living alone without kids at home)	219 (21%)	88 (19%)
Living alone with kids (Living alone with kids at home)	114 (11%)	47 (10%)
Partner but not living together (Have a partner but not living together)	80 (8%)	40 (8%)
		
Confidence, median (IQR)	6 (5;8)	6 (5;8)
Importance, median (IQR)	10 (9;10)	10 (9;10)
Knowledge, median (IQR)	5 (2;7)	5 (3;7)

^a^Calculated for participants who responded to question 1.

### Perspectives on intervention design and content

Table 2 shows the number of participants who selected each response option for all questions. Responses to questions 1–3 indicated that respondents overall reported that the support tool met their needs, that the content of the support tools was useful, and that the text messages were useful (for all: median = 4, IQR = 3;5). The median system useability score was 85 (IQR = 75;95), which can be interpreted as highly acceptable usability. The majority of respondents indicated that, if they were to continue using the support, they would do so for at least 1–2 months with several stating they would use it for another 6+ months. Most respondents also stated that they would recommend the support tool to a friend who wished to reduce their alcohol consumption.

**Table 2:** Response rates for each option in questions 1–5.

**Question**	**Response option**				
	**1 = Not very well/helpful**	**2**	**3**	**4**	**5 = Very well/helpful**
**Question 1 Suited to needs**	45 (9%)	56 (12%)	132 (28%)	115 (24%)	127 (27%)
**Question 2 Support tool useful**	22 (5%)	34 (7%)	129 (27%)	143 (30%)	151 (32%)
**Question 3 Text messages useful**	23 (5%)	36 (8%)	127 (27%)	131 (27%)	162 (34%)
	**1–2 months**	**3–6 months**	**6+ months**	**Don’t know**	**No**
**Question 4 Continued use**	67 (14%)	107 (22%)	151 (32%)	73 (15%)	81 (17%)
	**Yes**	**No**	**Don’t know**		
**Question 5 Recommend to friend**	378 (79%)	32 (7%)	69 (14%)		

OR and the probability of association for responding in more agreement to questions 1–3, determined with ordinal regression, are shown in Table 3. The results indicate that, all else being equal, individuals with lower total weekly consumption were more likely to respond with stronger agreement to all three questions (probability of association >99.9% for all) whilst frequency of heavy episodic drinking seemed to have less influence on responses. Higher baseline confidence was associated with an increased likelihood of stronger agreement with all three questions, as was higher baseline importance, in particular for rating the text messages as useful.

**Table 3:** OR of responding more agreeable on three questions regarding suitability of a digital alcohol intervention.

	**Question 1 Suited needs**		**Question 2 Support tool useful**		**Question 3 Text messages useful**	
	**Median** ^**a**^ **(95 CI)**	**Post. Prob** ^**b**^ **>/< null**	**Median** ^**a**^ **(95 CI)**	**Post. Prob** ^**b**^ **>/< null**	**Median** ^**a**^ **(95 CI)**	**Post. Prob** ^**b**^ **>/< null**
**Man vs. Woman**	1.27 (0.9; 1.78)	91.5%	0.85 (0.6; 1.2)	81.8%	1.19 (0.84; 1.68)	83.9%
**Age**	1.0 (0.99; 1.02)	55.3%	1.0 (0.98; 1.01)	60.1%	0.99 (0.98; 1.01)	79.3%
**Total weekly consumption**	0.97 (0.95; 0.98)	>99.9%	0.97 (0.95; 0.98)	>99.9%	0.97 (0.95; 0.99)	>99.9%
**Frequency of heavy episodic drinking**	1.01 (0.98; 1.03)	65.5%	1.01 (0.99; 1.04)	83.4%	1.02 (0.99; 1.05)	88.8%
**Confidence**	1.1 (1.03; 1.18)	99.5%	1.09 (1.02; 1.17)	99.6%	1.05 (0.98; 1.13)	92.4%
**Importance**	1.28 (1.13; 1.46)	>99.9%	1.16 (1.02; 1.32)	98.9%	1.27 (1.12; 1.45)	>99.9%
**Know-how**	0.94 (0.89; 1.0)	97.4%	0.97 (0.91; 1.03)	81.8%	0.98 (0.92; 1.04)	77.6%
**Living alone with kids at home** ^**c**^	0.87 (0.45; 1.64)	66.9%	1.1 (0.59; 2.1)	62.1%	0.75 (0.39; 1.43)	80.7%
**Have a partner but not living together** ^**c**^	0.65 (0.33; 1.3)	88.9%	0.87 (0.45; 1.71)	65.4%	0.7 (0.36; 1.43)	84.1%
**Living with somebody without kids** ^**c**^	0.74 (0.44; 1.23)	87.7%	1.11 (0.67; 1.83)	66.1%	0.78 (0.47; 1.3)	82.6%
**Living with somebody and kids** ^**c**^	0.82 (0.5; 1.31)	80.4%	1.04 (0.65; 1.66)	55.8%	0.67 (0.41; 1.09)	94.7%

^a^OR point estimate given by the median of the posterior distribution and 95% CI defined by the 2.5% and 97.5% percentiles of the posterior distribution.

^b^The proportion of the posterior distribution that is above or below the null (OR = 1) in the same direction as the median of the posterior distribution.

^c^Compared to living alone without kids.

Multiple linear regression indicated that, all else being equal, SUS scores were positively associated with confidence (median of posterior distribution = 0.56, probability of association = 97.7%) and importance (median of posterior distribution = 1.01, probability of association = 97.0%), and inversely associated with know-how (median of posterior distribution = −0.31, probability of association = 89.0%). No other baseline characteristics showed marked associations with SUS scores.

### Free-text responses

There were 191 comments, of which 25 were excluded from the qualitative analysis as they did not pertain to experiences of the intervention. Table 4 summarizes the categories and subcategories of positive- and negative-leaning comments detected.

**Table 4:** Identified (sub)categories coded as positive- and negative-leaning that were associated with experiences of the intervention.

	**Category**	**Sub-category**
**Positive**	Increased awareness and reflection on drinking behaviour (43)	
	Design (86)	Receiving reminders and messages was useful (45)
		The content (information and advice) was useful (24)
		System was supportive and motivating (9)
		Tracking drinking was useful (8)
**Negative**	Support perceived as impersonal (49)	Incompatibility—did not identify as target group for the intervention (13)
		Advice was not specific/individualized (12)
		Lack of social aspect/support (15)
		Underlying reasons for drinking not addressed (9)
	Design (70)	Features/questions confusing or flawed (31)
		Reminders repetitive or too frequent (22)
		Information too negative/not enough positive motivation (9)
		Advice insufficient or only surface level (8)

For both positive and negative aspects, design was the most coded for category. Positive design aspects mostly pertained to the perceived usefulness of the information and advice given, and the value of reminders. That the support was perceived as non-judgemental and imparted the feeling that individuals were in control of their own behaviour were also seen as positives. Tracking and seeing statistics about their drinking was often perceived as useful, although issues with recalling alcohol consumption during the past month were noted. Namely, reporting monthly consumption on a weekly basis was considered confusing, and difficulty remembering sometimes led to concerns that they were inadvertently being dishonest. The second category of positive aspects was participants’ recognition that the intervention increased their awareness of, and encouraged them to reflect on, their drinking behaviour. Notably, 35% of comments coded for highlighting the usefulness of receiving reminders and messages were also coded for mentioning an increased awareness of and reflection on drinking behaviour. This suggests that in several cases the reminders served as a direct prompt for evaluating one’s behaviour.

*‘Regular reminders means that you think about alcohol, the alcohol norm, regularly. That helps me be aware of casual drinking, to question whether alcohol is really necessary in all situations.’*

It was nonetheless noted that comparing current consumption to their own past behaviour may be more fruitful than comparing to an ideal situation. Related to reflection on drinking behaviour and its consequences, an aspect that was perceived negatively was the strong focus on the negative consequences of alcohol consumption at the cost of highlighting the benefits of reducing consumption or giving positive reinforcement when consumption is reduced.

*‘The will to drink less is ruined by reading about how bad it is/you are. Positive parts of reduced drinking would make you choose to hold on and keep going.’*

Several participants also suggested that individualization of some sort should be possible, potentially according to the reasons they drink or the consequences they experience from it. Relatedly, the intervention was sometimes experienced as impersonal, with participants mentioning that they would have appreciated more personal feedback or contact with another person.

*‘Alcohol problems look different for different individuals… the SMS messages are grounded in a specific problem, you should be able to choose what type of addiction you have.’*

Some respondents also reflected that the text messages were too frequent, too repetitive, or only offered surface level information. It was also highlighted by some that they felt like they did not fall into a presumed target group for the intervention, noting that it seemed targeted towards individuals with milder alcohol issues or younger people.

*‘Could best help someone who is on route to becoming addicted. It’s more difficult if you already are.’*

## Discussion

The aim of this study was to understand the experiences of the intervention group from an RCT regarding the support tool they were given access to. The results of this RCT have been previously reported, showing that the intervention was effective in reducing average drinking relative to information provision (Bendtsen *et al*. 2022). Here, we attempted to understand the elements of the support tool that were (un)helpful according to the intervention group, in order to improve future intervention design.

Taken together, the results indicated that respondents displayed a generally positive view towards the intervention: the majority responded that it was at least adequately well-suited to their needs, and that the content of the intervention and text messages were helpful. Those who were more likely to rate the support tool favourably overall (higher SUS score) were those who reported having higher baseline confidence in their ability to change and who considered change important, as well as those with lower self-reported knowledge on how to reduce their drinking and lower total weekly consumption. That some noted in the free-text responses that the advice seemed surface-level or offered nothing new supports this analysis, reinforcing that people who already feel like they have knowledge on how to change likely felt less satisfied with the support.

Analysis of the free-text responses revealed that key positive aspects of the intervention design were its perception as motivational, non-judgemental, accessible, and supportive. Regular reminders and tracking progress were also often regarded as positive, in agreement with previous work on mHealth app design indicating that monitoring, feedback, goal setting, rewards and prompts were beneficial for engagement, motivation, and imparting a sense of autonomy (Perski *et al*. 2017). Importantly, several individuals indicated not only that receiving reminders was useful but that these reminders acted as direct prompts to reflect on their drinking behaviour. This is in line with the mechanisms for behavioural change considered key during the development of the intervention (Bendtsen and McCambridge 2019), suggesting that the intervention largely worked as intended. Nonetheless, areas for improvement were also highlighted. Some individuals suggested that the overall look of the support tool could be altered, possibly taking inspiration from modern health and well-being apps, however this may speak to personal preferences rather than inherent design flaws. Several participants raised a desire for individualization or customisability in, for example, how changes in drinking habits are presented (e.g. comparing current consumption to their own previously tracked data instead of against an ideal situation). These suggestions are unsurprising, since the pool of individuals seeking help to change their drinking behaviour is likely to be highly heterogeneous, and so a one-size fits all approach is unlikely to fulfil the needs of nor be equally engaging for all.

Given that those with more confidence in their ability to change at baseline also reported greater satisfaction with the intervention, it is possible that some who described the lack of individualization as a design flaw may have instead experienced lower confidence and motivation to reduce their drinking, which they then rationalized post-hoc as dissatisfaction with the intervention design. Nonetheless, it has been suggested that the efficacy of eHealth and mHealth interventions could potentially be improved if they can be adjusted to suit the individual needs of users (Knox *et al*. 2019) and evidence suggests that customization and individualization can be beneficial in terms of adherence and engagement. For example, in a systematic review of factors affecting adherence to mHealth apps targeting prevention or management of non-communicable diseases (NCDs, personalization and tailoring of content to the user’s needs as well as individualized push notifications were found to have positive effects on adherence (Jakob *et al*. 2022). In another study, the content and frequency of text messages was tailored to participants’ baseline drinking patterns, which was reported to be an effective approach (Haug *et al*. 2013). Some participants here also specifically mentioned liking the optional module in the support tool where they could author their own prompts to be sent to them in the form of a text message at a later point in time, consistent with the desire for individualization.

Relatedly, some participants reflected that their underlying reasons or motives for drinking were not addressed by the intervention, leading to the support tool feeling impersonal or not going beyond ‘surface level’. Since increased awareness and reflection upon ones drinking behaviour was revealed as an important positive aspect, it seems that facilitating reflection upon the underlying reasons why an individual drinks, and tailoring the advice they receive accordingly, could be beneficial to future intervention design. Motives for drinking have been previously broadly categorized as positive (e.g. seeking pleasant feelings and social enhancement) and negative (e.g. relieving negative affect associated with anxiety or stress) reinforcement. These, in turn, have been associated with different levels and patterns of alcohol consumption (Kuntsche *et al*. 2005; Goldstein and Flett 2009).

One option could thus be to implement the short form of the Drinking Motives Questionnaire (Kuntsche and Kuntsche 2009) which measures the extent to which individuals’ motives for drinking are associated with Coping (e.g. reducing negative mood), Conformity (e.g. avoiding social consequences), Enhancement (e.g. improving positive mood), and Social (e.g. to fit in with a group or at social occasions) reasons. Taking these motives into account could offer an opportunity to fine-tune intervention content both in terms of information and suggested strategies for change. Tailoring advice towards individuals’ stage-of-change regarding alcohol consumption may also be valuable. Individuals in Action stages at post-treatment were found to be more likely to show reduced problematic drinking behaviour at 12-month follow-up than those in pre-Action stages (Heather *et al*. 2013). If stage-of-change is measured throughout a trial and intervention content dynamically changes to fit individual’s scores, it may be possible to both reduce the risk of repetitive reminders (regarded as a negative here) and offer the desired individualisation, whilst affecting overall intervention efficacy.

Both the qualitative and quantitative analysis indicated that participants who drank heavily at baseline or self-identified as very heavy or problem drinkers tended to describe the intervention as less suitable for their needs. Since the association between negative reinforcement and alcohol consumption may be stronger in those with alcohol dependence than those without (Cho *et al*. 2019), accounting for underlying motives may also improve the inclusivity of future interventions as well as their perceived suitability by heavier drinkers or self-identified problem drinkers. Advice that could bolster these individuals’ confidence in their ability to reduce their drinking may be particularly valuable, as confidence and readiness to change were associated with reduced heavy drinking days and increased abstinence days at 12-month follow-up amongst alcohol use disorder (AUD) patients (Gaume *et al*. 2017). Note that diagnoses such as AUD were not an aspect of the present work. Confidence in one’s ability to reduce drinking was also found to impact the likelihood of individuals randomized to the control group of the RCT the data analyzed here originate from to try to reduce drinking on their own (Gunnarsson *et al*. 2023). Ways to leverage intervention design and content in order to positively influence participants’ sense of confidence and motivation whilst attempting to change their drinking habits should therefore be considered. For example, here individuals sometimes mentioned wanting more positive reinforcement or information on the benefits of reducing alcohol consumption instead of repeated warnings about the negative consequences of drinking. However, increased awareness of the negative consequences of alcohol consumption may be a primary driver of reduced drinking (Müssener *et al*. 2018), and so such information should not be discarded entirely.

### Limitations

There are limitations with the present work that should be acknowledged. First, although the subset of intervention group individuals who responded to the user experience questionnaire seems to reflect the full intervention group quite well demographically (Table 1), it remains likely that survivorship bias could impact the results, both quantitative and qualitative. The data analyzed likewise cannot necessarily allow us to speak for individuals randomized to the intervention group who did not respond to these questions. Indeed, it is possible that individuals who found the intervention less suitable for their needs would be less likely to respond to the user experience survey, and data from these individuals could have affected the results. It should also be noted that the regression analyses were exploratory in nature, not pre-planned, and should be treated as such. Finally, we did not assess people’s expectations of the intervention prior to them taking part in the main trial. Since expectations of any product or tool can colour the experience of using them, better understanding of how (in)congruent participant experiences are with their expectations would be beneficial to future intervention design.

## Conclusions

The majority of respondents appeared satisfied with the support tool. Most rated the usefulness of the contents of the intervention and text messages favourably, reported that they would continue using it for at least 1–2 months, and stated that they would suggest it to a friend seeking help for their drinking. The intervention was found to be most satisfactory for those with higher confidence in their ability to reduce their drinking, who thought it was important to do so, and who drank less heavily at baseline. An important positive aspect of the intervention was that the reminders were often perceived as helpful and in several instances were reported as directly contributing to internal reflection on one’s drinking behaviour, supporting the theory underlying the intervention during its development. Other positives included that it was supportive without being judgemental, perceived as motivational, and that the information and advice were often helpful. The primary negatives included the repetitive nature of the reminders, that advice could be surface level, and that no individualization or customization was possible. Investigating the effectiveness of tailoring advice according to individuals’ motives for drinking or stage-of-change may be fruitful directions for future digital alcohol intervention trials.

## Author contributions

Elizabeth Collier (Formal analysis [Lead], Funding acquisition [Supporting], Methodology [Equal], Writing—original draft [Lead], Writing—review & editing [Lead]), Jenny Blomqvist (Formal analysis [Supporting], Writing—review & editing [Equal]), and Marcus Bendtsen (Conceptualization [Lead], Funding acquisition [Lead], Investigation [Lead], Methodology [Equal], Project administration [Lead], Validation [Lead], Visualization [Lead], Writing—review & editing [Equal])

## Conflict of interest statement

MB owns a private company (Alexit AB) that maintains and distributes evidence-based lifestyle interventions to be used by the public and in health care settings. Alexit AB played no role in developing the intervention, study design, data analysis, data interpretation, or writing of this report. Services developed and maintained by Alexit AB were used for sending text messages and data collection. ESC and JB declare no competing interests.

## Funding

This study was conducted under the auspices of the Alcohol Research Council of the Swedish Alcohol Retailing Monopoly (grant numbers 2019–0056 and 2020–0043) and the Swedish Research Council for Health, Working Life, and Welfare (grant number 2022–00193). The funders had no role in study design, data collection, data analysis, data interpretation, or writing of the report.

## Data availability

De-identified datasets generated during and/or analyzed during the current study will be made available upon reasonable request to the corresponding author, after approval of a proposal and with a signed data access agreement.
